# Mediating Effect of Infant Rapid Weight Gain on the Association Between Exclusive Breastfeeding and the Risk of Obesity Later in Life

**DOI:** 10.1111/ijpo.70039

**Published:** 2025-07-14

**Authors:** Carlos Nagore González, Iris Iglesia Altaba, Cristina Guillén Sebastián, Maria Luisa Alvarez Sauras, Sheila García Enguita, Luis A. Moreno, Gerardo Rodríguez

**Affiliations:** ^1^ Instituto de Investigación Sanitaria Aragón (IISAragón) Zaragoza Spain; ^2^ Primary Care Interventions to Prevent Maternal and Child Chronic Diseases of Perinatal and Developmental Origin Network (RICORS), RD21/0012/0012, Instituto de Salud Carlos III Madrid Spain; ^3^ Growth, Exercise, Nutrition and Development (GENUD) Research Group and Instituto Agroalimentario de Aragón (IA2) University of Zaragoza Zaragoza Spain; ^4^ CIBER Fisiopatología de la Obesidad y Nutrición, Instituto de Salud Carlos III Madrid Spain

**Keywords:** body mass index, breastfeeding, infant feeding, obesity, rapid weight gain

## Abstract

**Introduction:**

Obesity is a global health problem, with an impact on future health. Early factors such as infant feeding and rapid weight gain (RWG) may play a role in its development. However, their contribution is not fully understood.

**Objective:**

To analyse associations and potential interactions between the type of infant feeding during the first 4 months of age, RWG and later development of obesity in children at 6 years of age.

**Materials and Methods:**

This observational longitudinal study examines growth in 834 children born at term with adequate weight for their gestational age. Data were collected from birth to 6 years, focusing on weight, height and body mass index (BMI) z‐scores. The relationships between infant feeding, RWG, and BMI at 6 years were explored using mediation models.

**Results:**

Formula‐fed infants showed higher BMI z‐scores and obesity prevalence at 6 years compared to exclusively breastfed infants (*p* = 0.012 and *p* = 0.003 respectively). RWG also was associated with higher BMI z‐scores and obesity (*p* < 0.001 in both cases), with formula‐fed infants more likely to experience RWG (39.7% vs. 32.2%; *p* = 0.036). Mediation analysis revealed that the association between feeding type and BMI at 6 years is partly positively mediated by infant RWG up to 6 months.

**Conclusions:**

Infant feeding practices, particularly exclusive breastfeeding in the first 4 months, are associated with weight and BMI trajectories up to 6 years. Rapid weight gain mediates this relationship in the first 6 months, but from 6 to 12 months.

## 1 Introduction

Obesity among children and adolescents is a relevant health problem, with global prevalence continuing to rise. According to World Health Organisation (WHO) data, since 1990, the number of children and adolescents experiencing obesity has quadrupled. By 2022, an estimated 37 million children under the age of 5 and more than 390 million children and adolescents aged 5–19 were living with overweight or obesity, including 160 million with obesity. Almost half of children under 5 with overweight or obesity in 2022 resided in Asia [1].

Obesity, defined by WHO as the excessive accumulation of adipose tissue that may harm health, is a growing concern worldwide due to its association with various comorbidities such as diabetes, hypertension, musculoskeletal diseases, and cardiovascular conditions [2]. Currently, body mass index (BMI) is widely accepted as an indirect method for assessing overweight and obesity among children over 2 years of age. For children older than 5 years, WHO defines overweight as a BMI greater than 1 standard deviation (SD) and obesity when BMI exceeds 2 SD [1].

One of the early factors that appears to influence the development of obesity later in life is the type of infant feeding. Exclusive breastfeeding remains the optimal method of feeding infants during the first 6 months of life [3]. Recent studies have shown that formula‐fed infants have a significantly higher risk of developing obesity compared to those who were exclusively breastfed, with breastfeeding being considered a protective factor against increased BMI [4, 5]. However, the impact of breastfeeding on the child's future body composition seems to be influenced by genetic, environmental and socioeconomic factors [6, 7, 8]. Another main determinant that can significantly influence long‐term body composition in children is rapid weight gain during infant life. Various studies link rapid weight gain in the first months of life with an increased probability of obesity by school age and adolescence [9, 10, 11], even among term‐born infants with appropriate birth weight for gestational age [12].

Thus, both the type of infant feeding and the early growth pattern influence future risk of obesity and can be partially explained by the phenomenon of ‘metabolic and body composition programming’ that occurs due to changes in the nutritional environment during the first months of life [13]. Specifically, rapid weight gain in the early postnatal period could induce alterations in energy balance and appetite regulation, predisposing the individual to excessive body mass accumulation later in life [14]. Moreover, the rapid weight gain during the first 12 months of life, particularly formula feeding, has been associated with greater weight gain during the first year [15, 16, 17], likely due to higher protein and energy intake and poorer control over satiety‐related mechanisms [8]. The complexity of their interactions raises challenges in identifying which factors carry the most weight in developmental programming. This uncertainty highlights the need for more specialised analytical approaches to disentangle these intricate relationships.

Understanding the underlying mechanisms and factors that contribute to the long‐term health impact of breastfeeding and rapid childhood weight gain is essential. However, there is little scientific literature that analyses the effect of rapid weight gain in healthy full‐term infants with adequate weight on the risk of future obesity and, above all, the role played by the type of infant feeding in this possible relationship. This article aims to analyse associations between the type of infant feeding during the first 4 months, postnatal infant rapid weight gain and the development of obesity at 6 years of age in a term newborn population with adequate weight for the gestational age, with the purpose of identifying the individual role of each of these factors and their potential interaction on this phenomenon.

## 2 Materials and Methods

This study was designed following the guidelines of the STROBE initiative (Strengthening the Reporting of Observational Studies in Epidemiology) for observational studies [18].

### 2.1 Study Design and Population

‘Growth and Feeding during Infancy and Childhood in Children from Aragón’ (CALINA by its acronym in Spanish) is a representative cohort study of children born between March 2009 and February 2010 in Aragón, Spain. Participants were recruited in primary healthcare centers where trained paediatric personnel conducted the measurements. Once families agreed to participate in the study, prenatal data and birth characteristics were obtained from the medical records of the mothers and their newborns, as well as from direct interviews with families. Perinatal information was collected after enrollment, and the children were periodically re‐examined in primary care centers at 1, 2, 4 and 6 months; and at 1, 2, 3, 5 and 6 years. During each evaluation, in addition to the clinical assessment of health indicators, trained personnel measured growth indicators (weight and length/height depending on the age of the patient) to ensure consistency across measurements.

For this study, we included data from term‐born infants with appropriate birth weight for gestational age. Data were collected from newborns in Aragón, Spain, between March 2009 and February 2010. Only newborns whose families consented to participation were included in this study. Exclusion criteria included the presence of malformations, diseases, and physical disabilities. Preterm newborns or those born post‐term (gestational age less than 37 weeks or greater than 41 weeks), as well as those with a birth weight greater than 4000 g or less than 2500 g, were excluded. Subjects without the primary study variables (type of feeding up to 4 months, weight gain in the first year, and BMI z‐score at 6 years) were also excluded.

### 2.2 Measurement of Study Variables

Body weight was measured with a precision of 10 g using a scale/bioimpedance device (Tanita BC‐418, Corporation of America Inc., IL, USA). Length was measured in a supine position using a measuring board for children under 2 years. For children older than 2 years, height without shoes was measured to a precision of 0.1 cm while standing, using light clothing and a stadiometer (SECA 225, SECA Hamburg, Germany). The height and weight measurements were performed by trained personnel. BMI, weight, and height‐for‐age and sex z‐scores were calculated using Anthro v3.2.2 and Anthro Plus v1.0.4 software from WHO according to growth references.

#### 2.2.1 Body Mass Index at 6 Years

The sample was categorised based on BMI z‐scores (kg/m^2^) at 6 years into children with normal weight (z‐score below 1 SD), children with overweight (z‐score between 1 and 2 SD), and children with obesity (z‐score above 2 SD). Subsequently, the classification was grouped into children with obesity and children without obesity. This binary classification allowed for simplifying analyses and facilitating interpretation of the results.

In addition to the primary analysis based on BMI z‐scores, a supplementary analysis was performed using raw BMI values. This complementary approach aimed to address the well‐documented limitations of BMI z‐scores, including their reduced sensitivity to detect changes at the extremes of the BMI distribution and their dependence on specific reference populations.

#### 2.2.2 Rapid Weight Gain

We calculated the variation in weight z‐scores from birth to 1 year of age. The weight z‐score variation, as a continuous variable, was quantified by subtracting one from the other between each studied time period. Rapid weight gain (RWG) was coded as a dichotomous variable. RWG is defined as a positive change in the weight‐for‐age z‐score greater than 0.67 between two different ages during infancy [14]. In this case, it refers to changes between birth, 6 months, and 12 months of age. The z‐score values for weight‐for‐age at birth, at 6 months, and at 12 months were determined using the WHO Anthro software, in accordance with the WHO growth standards from 2006 to 2007, for both girls and boys.

#### 2.2.3 Infant Feeding Type

Children were classified according to the type of feeding received during the first 4 months of life into two categories: formula feeding and exclusive breastfeeding. The first group included infants who received formula regularly at any time in the first 4 months, either exclusively or combined with breastfeeding. The second group included infants who were exclusively breastfed up to 4 months.

### 2.3 Covariates

Demographic and perinatal variables of the sample population were collected, such as the family's place of residence, maternal education level, paternal and maternal BMI, maternal tobacco consumption during pregnancy, adequate gestational control, maternal age, gestational age, type of delivery, and newborn sex and anthropometric measures (weight, head circumference and length).

### 2.4 Statistical Analysis

Statistical analyses were performed using IBM SPSS Statistics (IBM SPSS Statistics for Windows, Version 29.0. Armonk, NY: IBM Corp.). A descriptive analysis was conducted using mean and standard deviation for continuous variables and frequencies and percentages for categorical variables.

Graphical representation of the means of the main anthropometric variables (weight z‐score, height z‐score, and BMI z‐score) at different ages was constructed using line graphs for each variable, divided by the type of feeding received in the first 4 months and by the existence of RWG during the first year. A linear mixed‐effects model was used to assess the impact of rapid weight gain, type of feeding, and follow‐up time on child growth. Raw BMI and main anthropometric z‐scores were analysed as dependent variables, showing significant effects of rapid weight gain and time, but not feeding type. The mean BMI z‐scores at 6 years in each group were represented as bar graphs with error bars. A *t*‐student test was used to analyse differences between BMI and z‐scores means at 6 years. Differences in the proportion of patients with obesity at 6 years between groups (depending on the type of feeding received during the first 4 months and the existence of RWG during the first year) were analysed with chi‐square tests, and the risk was assessed using an odds ratio (OR).

To evaluate whether the association between the type of feeding and BMI z‐score at 6 years was mediated by RWG, mediation models were examined using the PROCESS 4.2 macro for SPSS. Infant feeding was established as the independent variable (X), BMI z‐score at 6 years as the dependent variable (Y), and RWG as the moderating variable (M), analysed from birth to 1 year and subsequently from 0 to 6 months and from 6 to 12 months. In PROCESS, the statistical model used was Model 4 for simple mediations [19]. Indirect effects were calculated using the bootstrap method, a resampling technique that estimates the distribution of an indirect effect from the available data without making strong assumptions about the underlying data distribution, using a bias‐corrected bootstrapping procedure (10 000 samples). If the 95% confidence interval did not include zero, it indicated that the mediation effect was significant. This analysis was performed twice to identify any factors that could influence the relationship between the study variables, first without covariates and then including them (maternal education level, paternal and maternal BMI, and maternal smoking during pregnancy).

## 3 Results

From the initial sample (*N* = 1615), preterm newborns or those born post‐term (gestational age less than 37 weeks or greater than 41 weeks), as well as those with a birth weight greater than 4000 g or less than 2500 g, were excluded (*N* = 307), reducing the sample size to 1308. Additionally, subjects without the primary study variables (type of feeding up to 4 months, weight gain in the first year, and BMI z‐score at 6 years) were excluded (*n* = 474), resulting in a final sample of 834 children.

The characteristics of the sample, according to the type of feeding and the occurrence of RWG, are presented in Table 1 (qualitative variables) and Table 2 (quantitative variables). In order to avoid bias, after excluding newborns from the study due to missing data in one or more of the variables under investigation, the main descriptive variables of the excluded group were compared with those of the selected sample. No significant differences were found between the two groups, indicating that the exclusion did not impact the overall representativeness of the study sample.

**TABLE 1:** Qualitative variables of the study sample.

		Total % (*N*)	Exclusive breastfeeding	Formula feeding	No rapid weight gain	Rapid weight gain
Total % (*N*)		834	32.4 (270)	67.6 (564)	62.7 (523)	37.3 (311)
Gestational control	Yes	98.7 (823)	98.1 (265)	98.9 (558)	98.5 (515)	99 (308)
	No	1.3 (11)	1.9 (5)	1.1 (6)	1.5 (8)	1 (3)
Smoking during pregnancy	Yes No	18.3 (153) 81.7 (681)	12.2 (33) 87.8 (237)	21.3 (120)* 78.7 (444)*	15.7 (82) 84.3 (441)	22.8 (71)* 77.2 (240)*
Maternal education level	None Middle Hight	2.3 (19) 59.3 (495) 36 (300)	3 (8) 53 (143) 41.1 (111)	2 (11) 62.4 (352) 33.5 (189)	2.3 (12) 57.9 (303) 38 (199)	2.3 (7) 61.8 (192) 32.5 (101)
Type of delivery	Vaginal birth	79.4 (662)	87.4 (236)	75.5 (426)*	79.9 (418)	78.5 (244)
	Caesarean	20.6 (172)	12.6 (34)	24.5 (138)*	20.1 (105)	21.5 (67)
Sex	Male	51.4 (429)	49.3 (133)	52.5 (296)	50.7 (265)	52.7 (164)
	Female	48.6 (405)	50.7 (137)	47.5 (268)	49.3 (258)	47.3 (147)
Feeding type	Formula feeding	67.6 (564)	—	—	65 (340)	72 (224)*
	Exclusive breatfeeding	32.4 (270)	—	—	35 (183)	28 (87)*
Rapid weight gain	Yes	37.3 (311)	32.2 (87)	39.7 (224)*	—	—
	No	62.7 (523)	67.8 (183)	60.3 (340)*	—	—
BMI at 6 years	Normal weignt and overweight	85.5 (713)	90.7 (245)	83 (468)*	90.2 (472)	77.5 (241)*
	Obesity	14.5 (121)	9.3 (25)	17 (96)*	9.8 (51)	22.5 (70)*

*Note*: BMI: Body mass index.

* Chi‐2: *p* < 0.05 for the control group (formula feeding versus exclusive breastfeeding and rapid weight gain versus no rapid weight gain).

**TABLE 2:** Quantitative variables of the study sample.

	Total	Exclusive breastfeeding	Formula feeding	No rapid weight gain	Rapid weight gain
	Mean (SD)				
Paternal BMI	26.24 (3.42)	26.36 (3.46)	26.19 (3.4)	25.97 (3.34)	26.69 (3.51)*
Maternal BMI	23.46 (4.15)	23.27 (3.54)	23.55 (4.4)	23.25 (4.06)	23.81 (4.27)
Mothers’ age	32.03 (4.98)	32.79 (4.87)	31.62 (4.99)*	32.12 (4.96)	31.88 (5.01)
Gestational age	39.26 (1.19)	39.34 (1.16)	39.22 (1.2)	39.45 (1.14)	38.94 (1.19)*
Birth weight	3218 (356)	3296 (326)	3255 (369)	3375 (322)	3088 (339)*
Birth lenght	49.92 (1.73)	50.17 (1.69)	49.8 (1.74)*	50.25 (1.61)	49.36 (1.79)*
Head circumference	34.43 (1.28)	34.49 (1.29)	34.41 (1.28)	34.55 (1.27)	34.24 (1.27)*
Rapid weight gain z‐score (0–12 months)	0.39 (1.03)	0.24 (0.97)	0.46 (1.05)*	—	—
BMI z–score at 6 years	0.64 (1.28)	0.48 (1.12)	0.71 (1.34)*	0.38 (1.16)	1.08 (1.35)*

*Note*: BMI: Body mass index. SD: standard deviation.

* *t*‐student: *p* < 0.05 for control group (formula versus breast feeding and rapid weight gain versus no rapid weight gain).

### 3.1 Growth Patterns, BMI Z‐Score, and Obesity at 6 Years Depending on Infant Feeding Type

**FIGURE 1:**
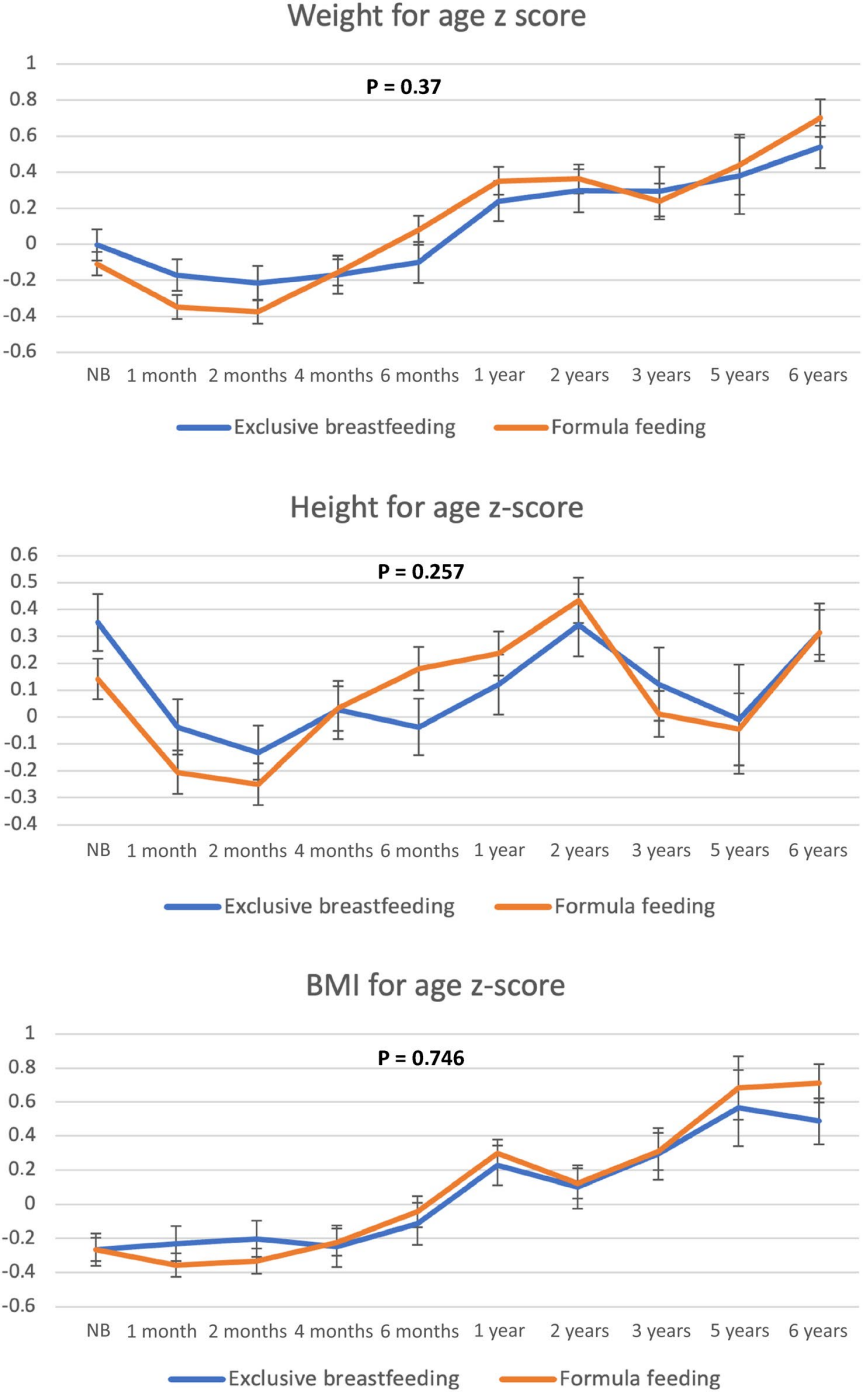
Growth trajectories according to type of infant feeding during the first 4 months of life. *p*‐value reflects the results obtained from the linear mixed‐effects model analysis.

**FIGURE 2:**
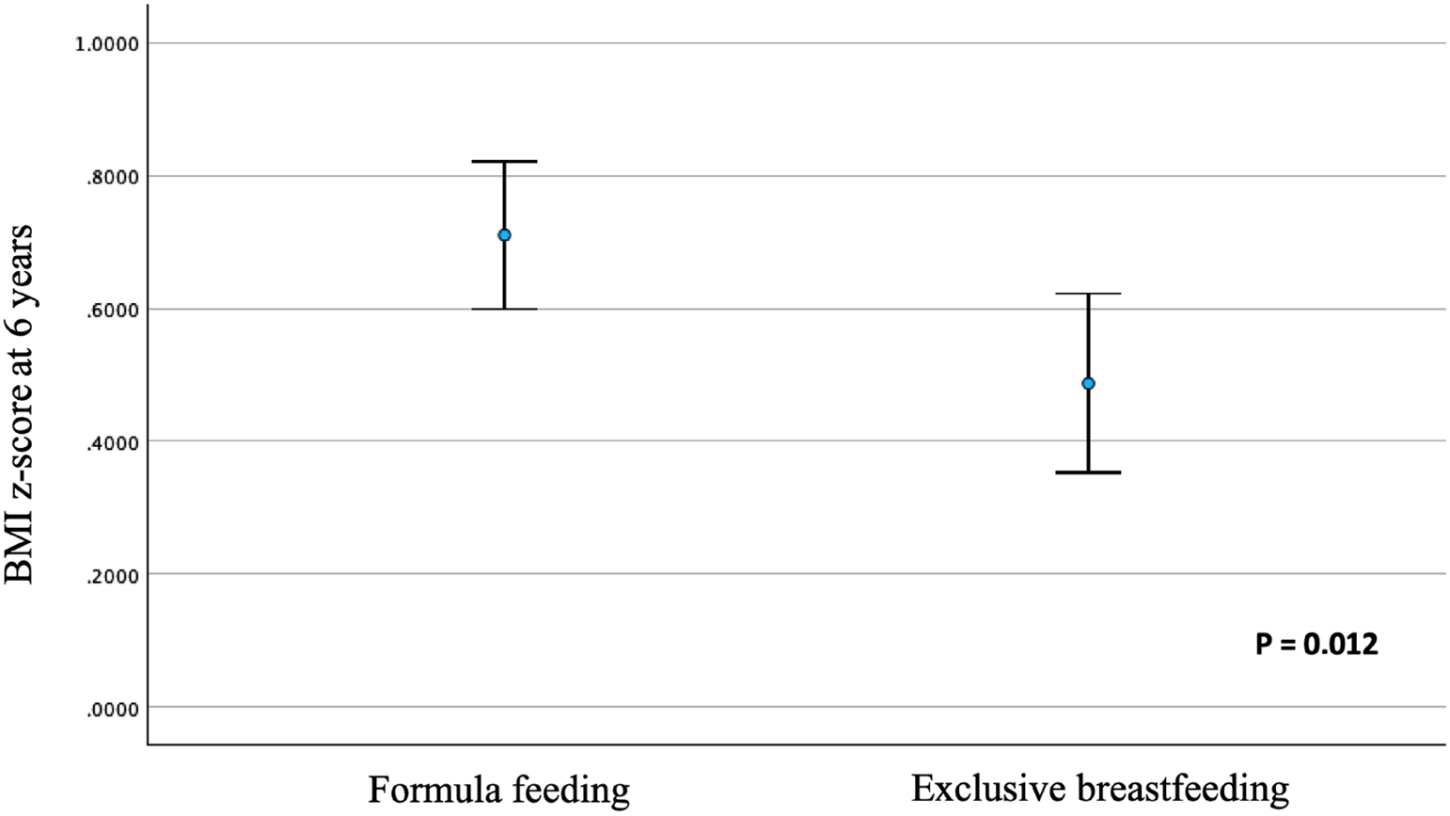
BMI z‐score at 6 years of age according to the type of infant feeding in the first 4 months of life.

The growth trajectories depending on infant feeding type during the first 4 months of life (Figure 1) showed no significant differences between formula‐fed infants and those who were exclusively breastfed. Formula‐fed infants had higher mean weight and BMI z‐scores at 6 years compared to those who were breastfed (*p* = 0.047 and *p* = 0.012, respectively) (Figures 1 and 2); these findings are confirmed by the analysis using raw BMI values (Figure S1). The percentage of children with obesity at 6 years was significantly higher in the formula‐fed group than in the exclusively breastfed group (97% vs. 83%; OR: 2.01 [95% CI: 1.26–3.2]; *p* = 0.003).

### 3.2 Growth Patterns, BMI Z‐Score and Obesity at 6 Years Depending on Infant RWG

**FIGURE 3:**
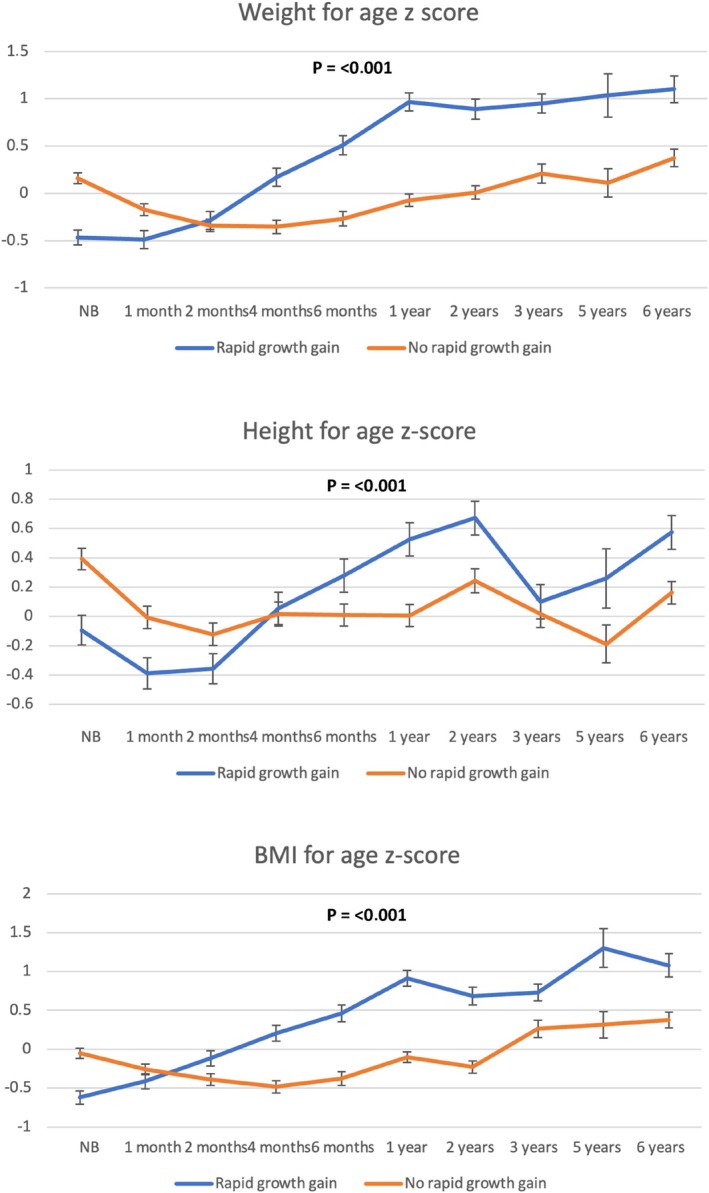
Growth trajectories according to rapid gain in weight or not during the first year of age. *p*‐value reflects the results obtained from the linear mixed‐effects model analysis.

**FIGURE 4:**
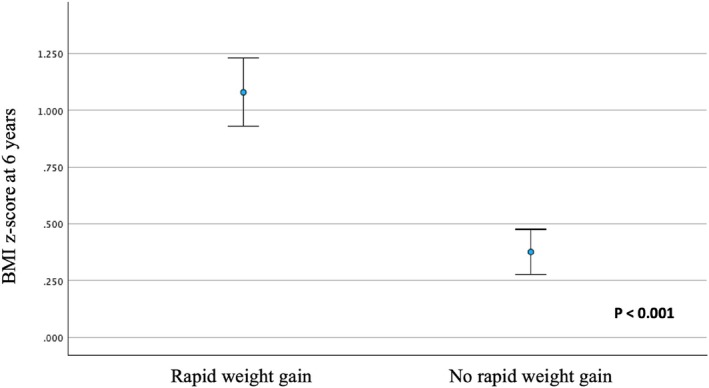
BMI z‐score at 6 years according to rapid weight gain or not during the first year of life.

Infants with RWG during the first year had higher weight, height and BMI z‐scores at 6 years compared to those who were breastfed (*p* < 0.001 in all cases) (Figures 3 and 4), and also showed distinct growth curve patterns throughout the follow‐up period. The analysis with raw BMI values supports these findings (Figure S2). The percentage of children with obesity at 6 years was significantly higher among those who experienced RWG during the first year compared to those who did not (22.5% vs. 9.8%; OR: 2.69 [95% CI: 1.82–3.98]; *p* < 0.001).

### 3.3 Relationship Between Feeding Type and Infant RWG

**FIGURE 5:**
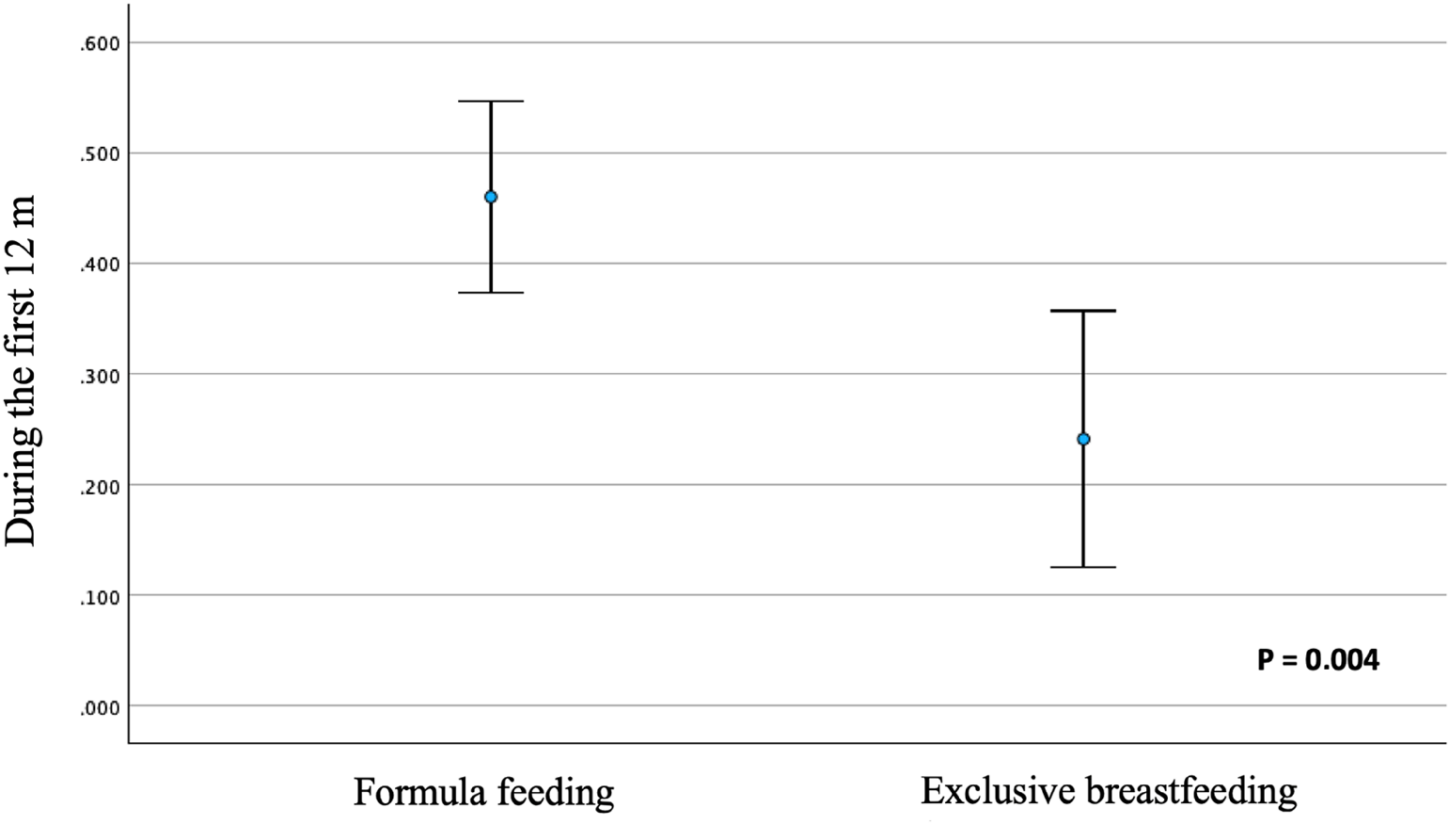
Weight gain (z‐score) during the first year of life according to the type of infant feeding in the first 4 months.

Figure 5 presents the comparison of mean weight gain (z‐score) during the first year of life depending on the type of feeding received in the first 4 months. Formula‐fed infants had a higher mean weight gain during the first year than those who were exclusively breastfed (*p* = 0.004). The percentage of infants who experienced RWG during the first year was significantly higher in the formula‐fed group than in the exclusively breastfed group (39.7% vs. 32.2%; OR 1.39 [95% CI: 1.02–1.88]; *p* = 0.036).

### 3.4 BMI Z‐Score and Obesity at 6 Years Grouped by Feeding Type and Infant RWG

**FIGURE 6:**
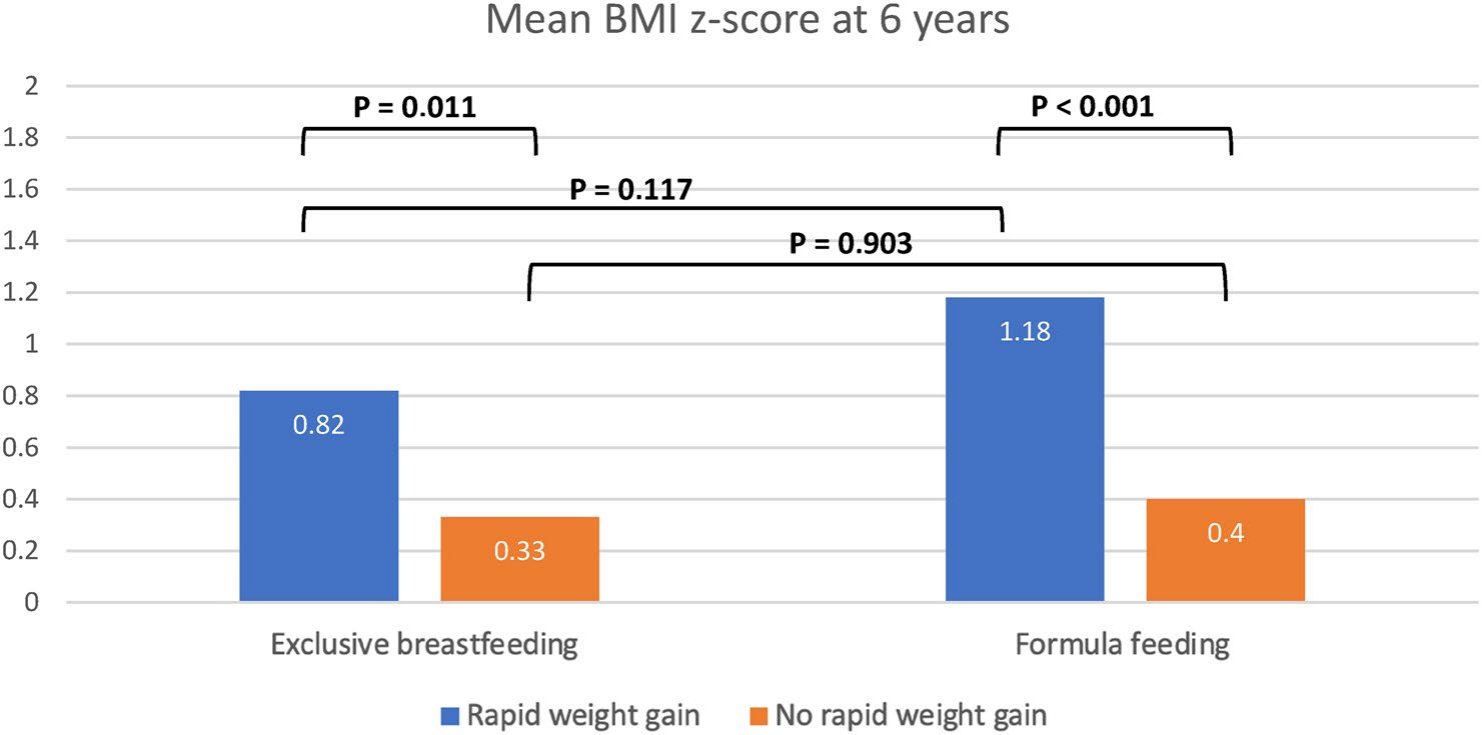
BMI z‐score at 6 years of age according to type of infant feeding and weight gain from 0 to 12 months of age.

**FIGURE 7:**
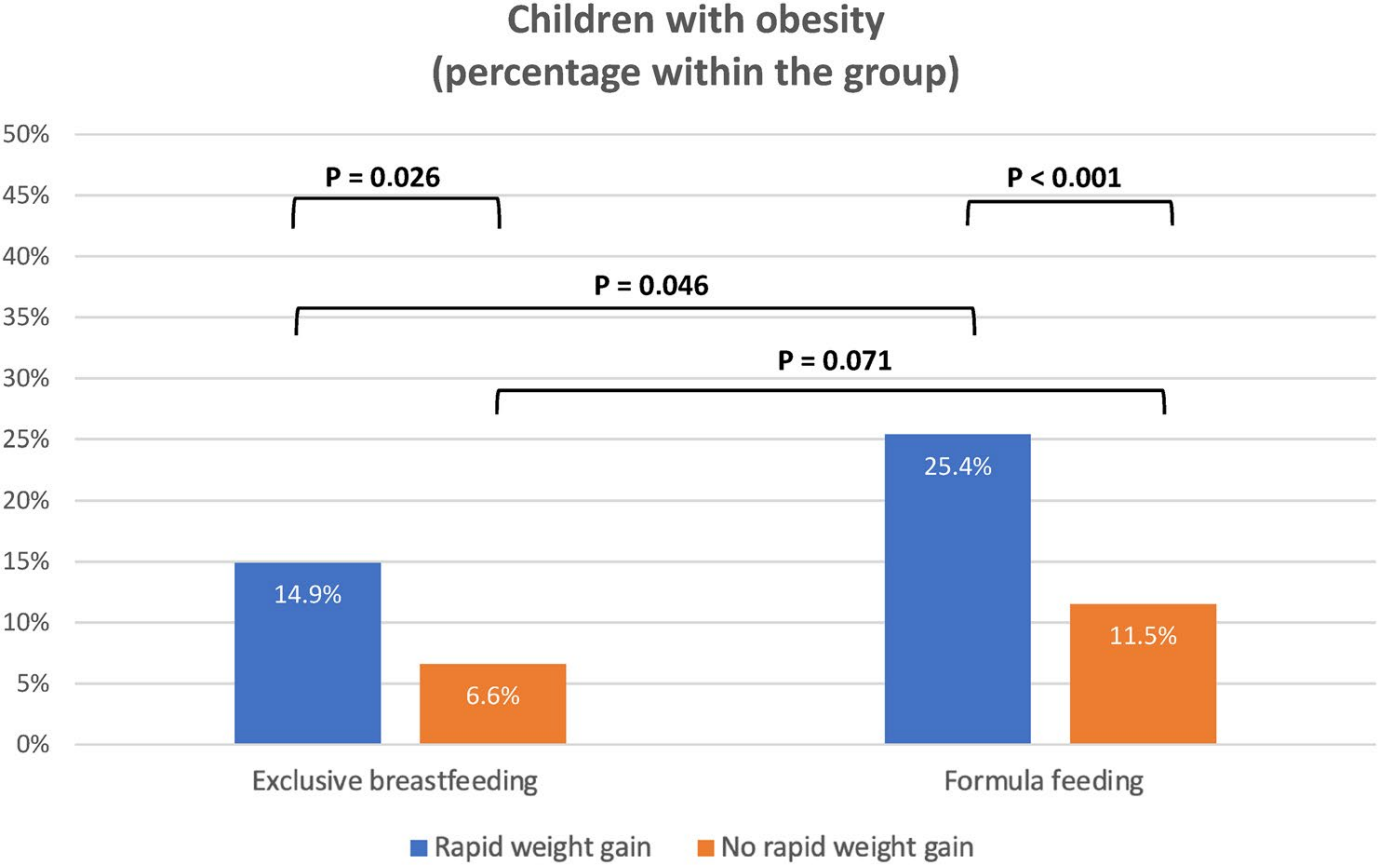
Percentage of children with obesity at 6 years according to type of infant feeding and weight gain from 0 to 12 months of age.

Figures 6 and 7 present the mean BMI z‐scores and the percentage of children with obesity at 6 years in the different groups according to the type of infant feeding in the first 4 months and RWG occurrence during the first year of life. Significant differences were observed within each group of feeding type depending on whether they underwent RWG or not. These differences were significant for mean BMI z‐scores at 6 years (both in the exclusive breastfeeding group and in the formula feeding group; *p* = 0.011 and *p* < 0.001, respectively) and also for the percentage of children with obesity at 6 years (both in the exclusive breastfeeding group: 14.9% vs. 6.6%, OR: 2.5, IC 95%: 1.09–5.75, *p* = 0.026; and in the formula feeding group: 25.4% vs. 11.5%, OR: 2.6, IC 95%: 1.68–4.13, *p* < 0.001). The differences in the percentage of children with obesity at 6 years were likewise significant, although to a lesser extent, between the groups with different feeding types that underwent RWG (25.4% in the formula‐fed group vs. 14.9% in the breastfeeding group, OR: 1.9, IC 95%: 1–3.77, *p* = 0.046). However, when RWG did not occur, there were no significant differences in obesity rates at 6 years between the feeding type groups (*p* = 0.071).

### 3.5 Mediation Study: Effect of Infant Feeding Type on the Relationship Between Rapid Infant Weight Gain and BMI Z‐Score at 6 Years

**FIGURE 8:**
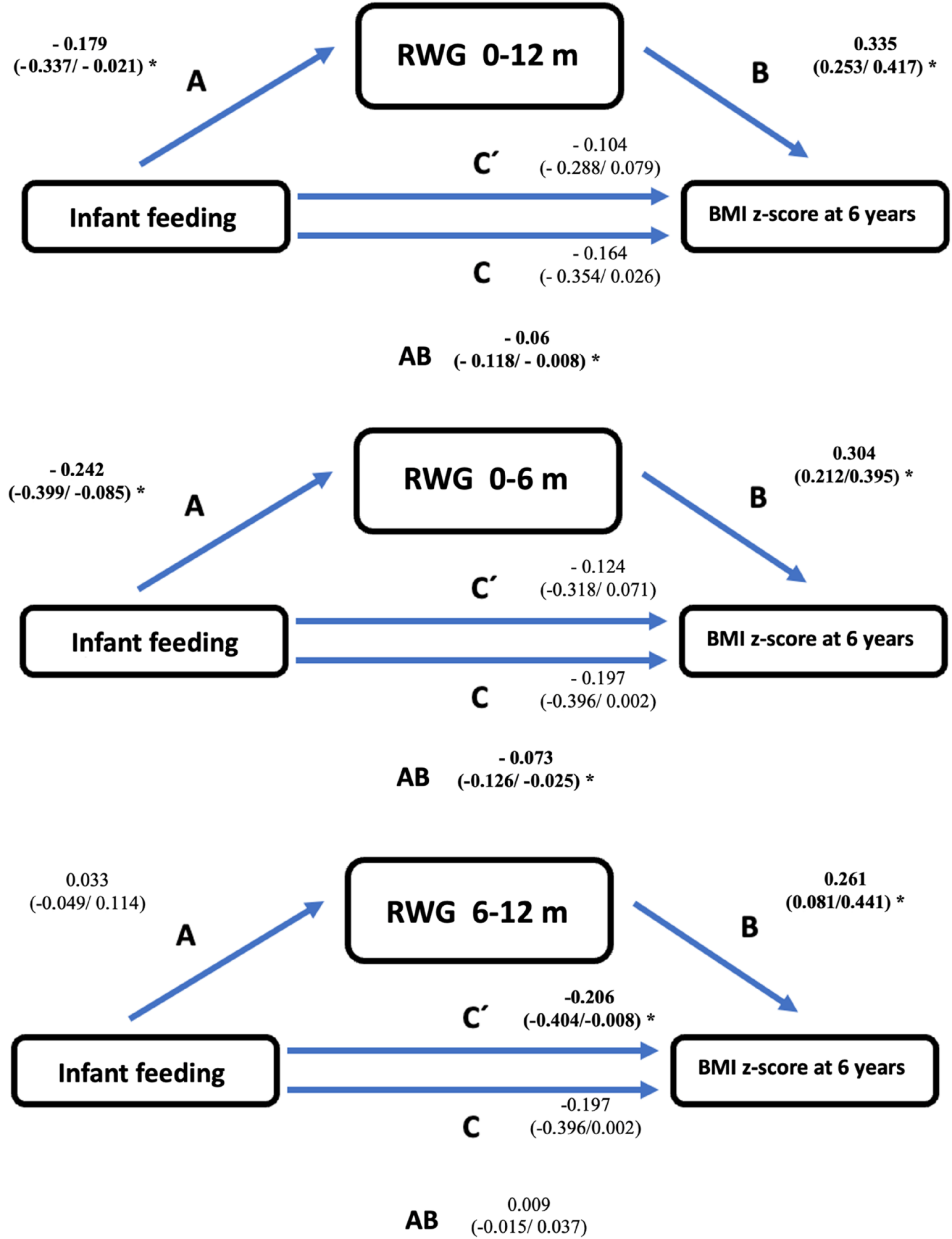
Graphical representation of the analysis of the mediation exerted by the type of breastfeeding on the relationship between rapid weight gain (between 0–12, 0–6 and 6–12 months) and BMI z‐score at 6 years. Covariates: Maternal and paternal BMI, smoking during pregnancy, and maternal education. Effect A: Effect of the type of infant feeding on rapid weight gain. Effect B: Effect of rapid weight gain on z‐score BMI at 6 years. Effect C: Total effect of the type of infant feeding on the z‐score BMI at 6 years. Effect C: The effect of lactation on the z‐score BMI at 6 years (without AB). Effect AB: Indirect effect of rapid growth on the association between lactation and BMI z‐score at 6 years. BMI z‐score: Body mass index z‐score; RWG: Rapid weight gain. Statistically significant results are shown in bold type. * Lower *p*‐value 0.05.

Figure 8 and Table 3 show the results of the mediation analysis, assessing the effect of feeding type on the relationship between RWG in different periods (0–12, 0–6 and 6–12 months) and the BMI z‐score at 6 years. The results were adjusted for the following covariates: paternal and maternal BMI, maternal smoking during pregnancy, and maternal education level. In the unadjusted analysis (Rows A, Table 3), infant feeding during the first 4 months had a significant effect on infant RWG (Effect A) in all cases except for RWG between 6 and 12 months. On the other hand, RWG in all periods examined was significantly associated with the BMI z‐score at 6 years (Effect B). Moreover, infant feeding type during the first 4 months showed a significant direct association (C′) with the BMI z‐score at 6 years, but only when RWG between 6 and 12 months was considered as the mediator. In contrast, the indirect effect of infant feeding type on the BMI z‐score at 6 years, mediated by RWG (Effect AB), was not significant in this group.

**TABLE 3:** Mediation analysis of rapid growth between 0 and 12 months, 0–6 months and 6–12 months, infant feeding type in the first 4 months, and BMI z‐score at 6 years.

	Effect A (CI 95%)	Effect B (CI 95%)	Effect C (CI 95%)	Effect C′ (CI 95%)	Effect AB (CI 95%)	Mediation
Mediation of rapid growth from 0 to 12 months						
A (N: 834)	−0.219 (−0.367/−0.070)*	0.372 (0.291/0.453)*	−0.223 (−0.409/−0.038)*	−0.142 (−0.320/0.036)	−0.081 (−0.142/−0.027)*	+
B (N: 764)	−0.179 (−0.337/−0.021)*	0.335 (0.253/0.417)*	−0.164 (−0.354/0.026)	−0.104 (−0.288/0.079)	−0.06 (−0.118/−0.008)*	+
Mediation of rapid growth from 0 to 6 months						
A (N: 773)	−0.267 (−0.415/−0.118)*	0.346 (0.256/0.435)*	−0.249 (−0.444/−0.055)*	−0.157 (−0.346/0.032)	−0.092 (−0.151/−0.041)*	+
B (N: 709)	−0.242 (−0.399/−0.085)*	0.304 (0.212/0.395)*	−0.197 (−0.396/0.002)	−0.124 (−0.318/0.071)	−0.073 (−0.126/−0.025)*	+
Mediation of rapid growth from 6 to 12 months						
A (N: 773)	0.026 (−0.053/0.105)	0.225 (0.053/0.398)*	−0.249 (−0.444/−0.055)*	−0.255 (−0.445/−0.061)*	0.006 (−0.015/0.029)	−
B (N: 709)	0.033 (−0.049/0.114)	0.261 (0.081/0.441)*	−0.197 (−0.396/0.002)	−0.206 (−0.404/−0.008)*	0.009 (−0.015/0.037)	−

*Note*: A: Simple mediation analysis without adjustment. B: Simple mediation analysis adjusted for maternal education, paternal and maternal BMI at birth, and maternal smoking during pregnancy. Effect A: Effect of infant feeding type on rapid weight gain. Effect B: Effect of rapid weight gain on BMI z‐score at 6 years. Effect C: Total effect of infant feeding type on BMI z‐score at 6 years. Effect C′: Direct effect (without AB) of feeding on BMI z‐score at 6 years. Effect AB: Indirect effect of rapid growth on the relationship between infant feeding and BMI z‐score at 6 years.

Abbreviations: BMI, Body Mass Index; CI, Confidence Interval; N, Sample size.

* Statistically significant results (*p* < 0.05).

After adjusting the mediation models for covariates (Rows B), the results were similar but quantitatively somewhat lower (Figure 8). The effect of feeding type on RWG (A), as well as the effect of RGW on BMI z‐score at 6 years (B), remained significant in the same groups as in the unadjusted analysis. Moreover, the direct effect of infant feeding type on BMI z‐score at 6 years (C′) also remained significant when RWG between 6 and 12 months was considered as the mediator. The indirect effect (AB) also remained significant in the same groups as before the covariate adjustment.

## 4 Discussion

The relationship between infant feeding and increased BMI in childhood has been extensively analysed in the scientific literature, along with the association between RWG and future cases of obesity in children. However, the interaction between these factors and their true impact on a child's growth and health remains a subject of debate. The exact influence of infant feeding and RWG during different stages of development, as well as the interaction between them, is still not fully understood [6, 20, 21, 22, 23]. One explanation for the discrepancies found in current literature may lie in the heterogeneity of the definitions used, with varying breastfeeding durations and definitions of RWG.

In our study, we chose to define exclusive breastfeeding as feeding until 4 months of age, before the introduction of complementary foods, and differentiated it from infants who received formula even partially. This approach aimed to isolate the effect that bottle‐feeding could have on weight gain and future BMI in children. Moreover, by selecting term newborns, we aimed to highlight the influence of RWG without it being explained by a ‘catch‐up’ effect, which has been described as a risk factor for obesity in preterm or low‐birth‐weight infants, likely due to the distribution of fat deposits and glucose metabolism [24, 25].

Our study presents growth charts (weight, height, and BMI) using z‐scores for paediatric populations from birth to 6 years, considering both the type of feeding received in the first 4 months and rapid weight gain in the first year. The use of z‐scores to assess height, weight, and BMI in children under 5 years is a strength of this article, as it provides a standardised method to assess growth according to WHO guidelines. Z‐scores offer a more precise evaluation by considering age and sex variations, allowing comparisons with international growth standards [26]. Although in the linear mixed‐effects model of growth trajectories only rapid weight gain during the first year and the time of measurement showed a statistically significant effect on the evolution of growth patterns, both formula feeding and RWG appear to be associated with higher weight and BMI in children at 6 years, as well as with a higher prevalence of obesity at this age. Additionally, feeding type and RWG are also interrelated, with children fed with formula exhibiting greater weight gain during the first year.

When analysing the three study variables (feeding type, RWG, and BMI at 6 years) together, we observed that the influence of rapid weight gain on increased BMI z‐score at 6 years appears to be more significant than the feeding type. There were no differences in BMI z‐scores at 6 years between groups with different feeding types but similar weight gain during the first year. Regarding the development of obesity in children at 6 years, breastfeeding acted as a protective factor in children with RWG, associating with a lesser proportion of obesity in this group compared to those fed with formula. Similar results were found in a study by Ejlerskov et al. (2015), which concluded that exclusive breastfeeding for 4–6 months attenuated the impact of early growth on BMI at 3 years [27].

The previously described relationships between feeding type, rapid growth, and increased BMI in children are complex and vary depending on the stage of development. In our mediation analysis, infant feeding, when RWG was measured during the first year and the first 6 months, showed no direct effect on BMI at 6 years after adjusting for parental BMI, maternal education, and smoking. However, the absence of a total effect does not preclude the presence of an indirect effect. In mediation analysis, an exposure can influence an outcome indirectly through a mediator, even if no total effect is observed. In this case, the indirect effect of infant feeding type through RWG on BMI at 6 years remained significant in these same groups. These results suggest that infant feeding in the first months of life may have an effect on future BMI mediated by RWG, perhaps influenced by familial and social factors. Conversely, between 6 and 12 months of age, the influence of RWG on BMI diminished until it was no longer significant, as did the effect of infant feeding type on RWG. However, during this period, infant feeding showed a direct and independent effect on BMI at 6 years. Two scenarios are thus proposed, in which the relationships between these three factors evolve and change with the child's development: (a) A first stage until 6 months of life, where the effect of infant feeding primarily influences RWG during the first year and indirectly affects BMI at 6 years; (b) A second stage between 6 and 12 months, where infant feeding no longer influences rapid growth but has a direct and independent effect on BMI at 6 years. This direct effect could align with the metabolic programming theory, suggesting that certain components of breastfeeding have epigenetic effects on infants, influencing future body composition and playing a significant role in appetite regulation through hormones like leptin [28, 29, 30, 31]. Perhaps in the second semester, aspects related to infant feeding that influence infant growth and the future risk of obesity are associated with both qualitative and quantitative aspects of complementary feeding.

It is also important to consider that the indirect effect of infant feeding on BMI at 6 years, mediated by RWG, may be influenced by factors beyond the composition of the milk itself. In a study conducted by Alison K. Ventura et al. (2020) with data from 1062 newborns, it was concluded that significant increases in bottle‐feeding during the first 6 months postpartum were directly associated with greater weight gain during the first year and indirectly, through this weight gain, with a higher risk of obesity at 6 years. This is likely related to early overfeeding [32].

The fact that many of the effects analysed in the mediation study diminished when covariates such as parental BMI, maternal education, and smoking were included indicates that these factors played a role in each of the pathways examined. This supports the hypothesis that maternal, genetic, environmental, and behavioural factors influence a children's growth and body composition in the future [33] and that these factors should be taken into account, for example, in the design and implementation of future interventions aimed at preventing the development of overweight and obesity in children.

## 5 Limitations

This study did not include co‐factors closely related to obesity development, such as dietary patterns, sedentary behaviours, physical activity, sleep duration, or family income. Furthermore, some measurements, such as maternal weight, height, and education level, were self‐reported, which could introduce bias. Additionally, the study did not account for specific feeding practices, such as scheduled vs. on‐demand feeding or the addition of cereals to bottles, which could be relevant in understanding their effect on growth and weight gain. Atlast, the study population is limited to a region in Spain, so the extrapolation of these results to other populations should be done cautiously, taking into account sociocultural and demographic differences. Future studies with more diverse populations that consider the wide range of factors involved in obesity development are necessary to confirm the findings of this study.

Another important limitation of this study is the use of z scores to assess weight gain and growth. Although z scores allow for standardised comparisons across different ages and populations, they may also result in a loss of intraindividual variability when analysing changes over time. This has been highlighted in the literature as a potential drawback of reference‐based scores, particularly in longitudinal analyses [34]. To reduce this limitation, a supplementary analysis was performed using mean raw BMI values.

## 6 Strengths

One of the strengths of this study is its comprehensive longitudinal design, which tracks a cohort of children from birth to 6 years of age, providing robust data on growth patterns. The study's use of standardised growth measures, such as z‐scores for weight, height, and BMI, ensures precise comparisons across individuals and groups. Furthermore, the analysis of the mediating role of rapid weight gain (RWG) on the relationship between infant feeding type and BMI at 6 years adds a novel dimension to understanding the developmental impact of early nutrition. By adjusting for covariates, the study enhances the reliability of its findings.

## 7 Conclusions

In conclusion, infant feeding practices and RWG during the first year of life have significant associations with weight and body mass index (BMI) trajectories at 6 years of age. These factors appear to be interrelated, with RGW in the first 6 months acting as a mediator in the relationship between infant feeding type in the first 4 months and increased BMI at 6 years. On the other hand, between 6 and 12 months of age, the association of infant feeding type in the first 4 months with future BMI seems to be direct and independent without intermediation of RWG. Exclusive breastfeeding during the first 4 months of age allows attenuating the association between infant RWG and the risk of obesity later in life.

## Author Contributions

All authors have significantly contributed to the development of this scientific article. They were involved in the conception and design of the study, data collection and analysis, as well as the writing and revision of the manuscript. All authors have reviewed and approved the final version of the manuscript.

## Ethics Statement

This study was conducted in accordance with the ethical guidelines of the 1964 Declaration of Helsinki. Parents or legal guardians provided written informed consent for participation. Ethical approval was obtained in June 2008 (P108/0021) from the Clinical Research Ethics Committee of Aragón (CEICA).

## Conflicts of Interest

The authors declare no conflicts of interest.

## Supporting information


**Figure S1.** Growth trajectories according to type of infant feeding during the first 4 months of life, *p*‐value corresponds to the comparison of mean BMI at age 6 between the two groups.
**Figure S2.** Growth trajectories according to rapid gain in weight or not during the first year of age, *p*‐value corresponds to the comparison of mean BMI at age 6 between the two groups.
