# Supplementary material for: Mediating Effect of Infant Rapid Weight Gain on the Association Between Exclusive Breastfeeding and the Risk of Obesity Later in Life
Source: Pediatr Obes. 2025 Jul 14;20(10):e70039. doi: 10.1111/ijpo.70039 (PMC12414580; doi:10.1111/ijpo.70039)
Supplement: Supplementary file 1 — Figure S1. Growth trajectories according to type of infant feeding during the first 4 months of life, p‐value corresponds to the comparison of mean BMI at age 6 between the two groups. Figure S2. Growth trajectories according to rapid gain in weight or not during the first year of age, p‐value corresponds to the comparison of mean BMI at age 6 between the two groups. [file IJPO-20-e70039-s001.docx]

**SUPPLEMENTARY MATERIAL**

**Figure 1:** Growth trajectories according to type of infant feeding during the first 4 months of life.

p-value corresponds to the comparison of mean BMI at age 6 between the two groups.

**Figure 2:** Growth trajectories according to rapid gain in weight or not during the first year of age.

p-value corresponds to the comparison of mean BMI at age 6 between the two groups.
